# Supplementary material for: Pyruvate Dehydrogenase Kinase 1 inhibition mediated oxidative phosphorylation enhancement in cartilage promotes osteoarthritis progression
Source: BMC Musculoskelet Disord. 2023 Jul 20;24:597. doi: 10.1186/s12891-023-06585-6 (PMC10357736; doi:10.1186/s12891-023-06585-6)
Supplement: Supplementary file 1 — Additional file 1. [file 12891_2023_6585_MOESM1_ESM.zip › KEGG.Gsea.1653622432464/KEGG_REGULATION_OF_ACTIN_CYTOSKELETON.html]

Details for gene set KEGG\_REGULATION\_OF\_ACTIN\_CYTOSKELETON[GSEA]

|  || Dataset | 55457\_collapsed\_to\_symbols.cls.cls #OA\_versus\_normal.cls.cls #OA\_versus\_normal\_repos |
| Phenotype | cls.cls#OA\_versus\_normal\_repos |
| Upregulated in class | normal |
| GeneSet | KEGG\_REGULATION\_OF\_ACTIN\_CYTOSKELETON |
| Enrichment Score (ES) | -0.22781363 |
| Normalized Enrichment Score (NES) | -1.0907667 |
| Nominal p-value | 0.26760563 |
| FDR q-value | 0.53031343 |
| FWER p-Value | 1.0 |
Table: GSEA Results Summary

  

Fig 1: Enrichment plot: KEGG\_REGULATION\_OF\_ACTIN\_CYTOSKELETON      
 Profile of the Running ES Score & Positions of GeneSet Members on the Rank Ordered List

  

| SYMBOL | TITLE | RANK IN GENE LIST | RANK METRIC SCORE | RUNNING ES | CORE ENRICHMENT || 1 | ARPC1A | actin related protein 2/3 complex subunit 1A [Source:HGNC Symbol;Acc:HGNC:703] | 47 | 1.003 | 0.0132 | No |
| 2 | PDGFC | platelet derived growth factor C [Source:HGNC Symbol;Acc:HGNC:8801] | 103 | 0.911 | 0.0243 | No |
| 3 | FGF14 | fibroblast growth factor 14 [Source:HGNC Symbol;Acc:HGNC:3671] | 427 | 0.695 | 0.0106 | No |
| 4 | RRAS | RAS related [Source:HGNC Symbol;Acc:HGNC:10447] | 505 | 0.658 | 0.0156 | No |
| 5 | PFN2 | profilin 2 [Source:HGNC Symbol;Acc:HGNC:8882] | 560 | 0.638 | 0.0221 | No |
| 6 | VAV1 | vav guanine nucleotide exchange factor 1 [Source:HGNC Symbol;Acc:HGNC:12657] | 601 | 0.622 | 0.0295 | No |
| 7 | PPP1CA | protein phosphatase 1 catalytic subunit alpha [Source:HGNC Symbol;Acc:HGNC:9281] | 603 | 0.622 | 0.0399 | No |
| 8 | PPP1CC | protein phosphatase 1 catalytic subunit gamma [Source:HGNC Symbol;Acc:HGNC:9283] | 744 | 0.583 | 0.0387 | No |
| 9 | MAPK3 | mitogen-activated protein kinase 3 [Source:HGNC Symbol;Acc:HGNC:6877] | 757 | 0.579 | 0.0475 | No |
| 10 | ITGB2 | integrin subunit beta 2 [Source:HGNC Symbol;Acc:HGNC:6155] | 827 | 0.562 | 0.0516 | No |
| 11 | PIP4K2C | phosphatidylinositol-5-phosphate 4-kinase type 2 gamma [Source:HGNC Symbol;Acc:HGNC:23786] | 939 | 0.530 | 0.0518 | No |
| 12 | FGF18 | fibroblast growth factor 18 [Source:HGNC Symbol;Acc:HGNC:3674] | 969 | 0.525 | 0.0583 | No |
| 13 | ARPC5 | actin related protein 2/3 complex subunit 5 [Source:HGNC Symbol;Acc:HGNC:708] | 1118 | 0.492 | 0.0550 | No |
| 14 | PIP5K1B | phosphatidylinositol-4-phosphate 5-kinase type 1 beta [Source:HGNC Symbol;Acc:HGNC:8995] | 1291 | 0.459 | 0.0492 | No |
| 15 | FGF23 | fibroblast growth factor 23 [Source:HGNC Symbol;Acc:HGNC:3680] | 1449 | 0.436 | 0.0442 | No |
| 16 | CYFIP2 | cytoplasmic FMR1 interacting protein 2 [Source:HGNC Symbol;Acc:HGNC:13760] | 1636 | 0.408 | 0.0365 | No |
| 17 | ARPC3 | actin related protein 2/3 complex subunit 3 [Source:HGNC Symbol;Acc:HGNC:706] | 1743 | 0.393 | 0.0347 | No |
| 18 | FGF9 | fibroblast growth factor 9 [Source:HGNC Symbol;Acc:HGNC:3687] | 1833 | 0.380 | 0.0341 | No |
| 19 | ITGA4 | integrin subunit alpha 4 [Source:HGNC Symbol;Acc:HGNC:6140] | 1854 | 0.379 | 0.0390 | No |
| 20 | PFN1 | profilin 1 [Source:HGNC Symbol;Acc:HGNC:8881] | 1863 | 0.377 | 0.0447 | No |
| 21 | CD14 | CD14 molecule [Source:HGNC Symbol;Acc:HGNC:1628] | 1928 | 0.369 | 0.0459 | No |
| 22 | CRK | "CRK proto-oncogene, adaptor protein [Source:HGNC Symbol;Acc:HGNC:2362]" | 2018 | 0.360 | 0.0450 | No |
| 23 | GNG12 | G protein subunit gamma 12 [Source:HGNC Symbol;Acc:HGNC:19663] | 2168 | 0.342 | 0.0390 | No |
| 24 | ARPC1B | actin related protein 2/3 complex subunit 1B [Source:HGNC Symbol;Acc:HGNC:704] | 2184 | 0.340 | 0.0436 | No |
| 25 | PIK3CB | "phosphatidylinositol-4,5-bisphosphate 3-kinase catalytic subunit beta [Source:HGNC Symbol;Acc:HGNC:8976]" | 2186 | 0.340 | 0.0492 | No |
| 26 | CRKL | "CRK like proto-oncogene, adaptor protein [Source:HGNC Symbol;Acc:HGNC:2363]" | 2283 | 0.329 | 0.0472 | No |
| 27 | MYL2 | myosin light chain 2 [Source:HGNC Symbol;Acc:HGNC:7583] | 2376 | 0.317 | 0.0453 | No |
| 28 | EGF | epidermal growth factor [Source:HGNC Symbol;Acc:HGNC:3229] | 2399 | 0.315 | 0.0489 | No |
| 29 | ITGA1 | integrin subunit alpha 1 [Source:HGNC Symbol;Acc:HGNC:6134] | 2421 | 0.311 | 0.0525 | No |
| 30 | ACTN2 | actinin alpha 2 [Source:HGNC Symbol;Acc:HGNC:164] | 2532 | 0.299 | 0.0489 | No |
| 31 | NCKAP1L | NCK associated protein 1 like [Source:HGNC Symbol;Acc:HGNC:4862] | 2560 | 0.296 | 0.0517 | No |
| 32 | ENAH | ENAH actin regulator [Source:HGNC Symbol;Acc:HGNC:18271] | 2665 | 0.284 | 0.0483 | No |
| 33 | MYL5 | myosin light chain 5 [Source:HGNC Symbol;Acc:HGNC:7586] | 2705 | 0.279 | 0.0500 | No |
| 34 | FGFR3 | fibroblast growth factor receptor 3 [Source:HGNC Symbol;Acc:HGNC:3690] | 2812 | 0.269 | 0.0462 | No |
| 35 | MYLPF | "myosin light chain, phosphorylatable, fast skeletal muscle [Source:HGNC Symbol;Acc:HGNC:29824]" | 2816 | 0.268 | 0.0505 | No |
| 36 | ITGB5 | integrin subunit beta 5 [Source:HGNC Symbol;Acc:HGNC:6160] | 2823 | 0.267 | 0.0545 | No |
| 37 | FGF20 | fibroblast growth factor 20 [Source:HGNC Symbol;Acc:HGNC:3677] | 2849 | 0.265 | 0.0570 | No |
| 38 | ARPC2 | actin related protein 2/3 complex subunit 2 [Source:HGNC Symbol;Acc:HGNC:705] | 2893 | 0.261 | 0.0580 | No |
| 39 | PDGFRA | platelet derived growth factor receptor alpha [Source:HGNC Symbol;Acc:HGNC:8803] | 2943 | 0.256 | 0.0585 | No |
| 40 | MYL12A | myosin light chain 12A [Source:HGNC Symbol;Acc:HGNC:16701] | 3039 | 0.246 | 0.0552 | No |
| 41 | WASF2 | WASP family member 2 [Source:HGNC Symbol;Acc:HGNC:12733] | 3101 | 0.239 | 0.0544 | No |
| 42 | CYFIP1 | cytoplasmic FMR1 interacting protein 1 [Source:HGNC Symbol;Acc:HGNC:13759] | 3277 | 0.223 | 0.0444 | No |
| 43 | ARHGEF4 | Rho guanine nucleotide exchange factor 4 [Source:HGNC Symbol;Acc:HGNC:684] | 3307 | 0.220 | 0.0458 | No |
| 44 | PIP4K2B | phosphatidylinositol-5-phosphate 4-kinase type 2 beta [Source:HGNC Symbol;Acc:HGNC:8998] | 3416 | 0.210 | 0.0409 | No |
| 45 | CHRM2 | cholinergic receptor muscarinic 2 [Source:HGNC Symbol;Acc:HGNC:1951] | 3473 | 0.205 | 0.0399 | No |
| 46 | ITGAE | integrin subunit alpha E [Source:HGNC Symbol;Acc:HGNC:6147] | 3522 | 0.200 | 0.0395 | No |
| 47 | SSH3 | slingshot protein phosphatase 3 [Source:HGNC Symbol;Acc:HGNC:30581] | 3707 | 0.185 | 0.0282 | No |
| 48 | EZR | ezrin [Source:HGNC Symbol;Acc:HGNC:12691] | 3817 | 0.174 | 0.0225 | No |
| 49 | RAC3 | Rac family small GTPase 3 [Source:HGNC Symbol;Acc:HGNC:9803] | 3849 | 0.171 | 0.0230 | No |
| 50 | PDGFD | platelet derived growth factor D [Source:HGNC Symbol;Acc:HGNC:30620] | 3850 | 0.171 | 0.0258 | No |
| 51 | PAK1 | p21 (RAC1) activated kinase 1 [Source:HGNC Symbol;Acc:HGNC:8590] | 3861 | 0.169 | 0.0279 | No |
| 52 | INS | insulin [Source:HGNC Symbol;Acc:HGNC:6081] | 3885 | 0.166 | 0.0289 | No |
| 53 | TIAM1 | TIAM Rac1 associated GEF 1 [Source:HGNC Symbol;Acc:HGNC:11805] | 3967 | 0.159 | 0.0252 | No |
| 54 | VAV3 | vav guanine nucleotide exchange factor 3 [Source:HGNC Symbol;Acc:HGNC:12659] | 4082 | 0.148 | 0.0187 | No |
| 55 | MYL10 | myosin light chain 10 [Source:HGNC Symbol;Acc:HGNC:29825] | 4125 | 0.144 | 0.0178 | No |
| 56 | MYH10 | myosin heavy chain 10 [Source:HGNC Symbol;Acc:HGNC:7568] | 4169 | 0.141 | 0.0168 | No |
| 57 | SLC9A1 | solute carrier family 9 member A1 [Source:HGNC Symbol;Acc:HGNC:11071] | 4221 | 0.135 | 0.0151 | No |
| 58 | PIP4K2A | phosphatidylinositol-5-phosphate 4-kinase type 2 alpha [Source:HGNC Symbol;Acc:HGNC:8997] | 4400 | 0.121 | 0.0031 | No |
| 59 | FGF1 | fibroblast growth factor 1 [Source:HGNC Symbol;Acc:HGNC:3665] | 4436 | 0.118 | 0.0024 | No |
| 60 | F2 | "coagulation factor II, thrombin [Source:HGNC Symbol;Acc:HGNC:3535]" | 4745 | 0.092 | -0.0203 | No |
| 61 | FGF8 | fibroblast growth factor 8 [Source:HGNC Symbol;Acc:HGNC:3686] | 4761 | 0.090 | -0.0200 | No |
| 62 | MYL9 | myosin light chain 9 [Source:HGNC Symbol;Acc:HGNC:15754] | 4939 | 0.077 | -0.0326 | No |
| 63 | FGF5 | fibroblast growth factor 5 [Source:HGNC Symbol;Acc:HGNC:3683] | 4990 | 0.074 | -0.0353 | No |
| 64 | DOCK1 | dedicator of cytokinesis 1 [Source:HGNC Symbol;Acc:HGNC:2987] | 4998 | 0.073 | -0.0346 | No |
| 65 | FGF16 | fibroblast growth factor 16 [Source:HGNC Symbol;Acc:HGNC:3672] | 5013 | 0.072 | -0.0345 | No |
| 66 | FGF13 | fibroblast growth factor 13 [Source:HGNC Symbol;Acc:HGNC:3670] | 5070 | 0.067 | -0.0378 | No |
| 67 | MYLK | myosin light chain kinase [Source:HGNC Symbol;Acc:HGNC:7590] | 5079 | 0.066 | -0.0373 | No |
| 68 | MYLK3 | myosin light chain kinase 3 [Source:HGNC Symbol;Acc:HGNC:29826] | 5294 | 0.048 | -0.0533 | No |
| 69 | CHRM4 | cholinergic receptor muscarinic 4 [Source:HGNC Symbol;Acc:HGNC:1953] | 5312 | 0.046 | -0.0539 | No |
| 70 | FGFR4 | fibroblast growth factor receptor 4 [Source:HGNC Symbol;Acc:HGNC:3691] | 5351 | 0.044 | -0.0561 | No |
| 71 | FGF7 | fibroblast growth factor 7 [Source:HGNC Symbol;Acc:HGNC:3685] | 5386 | 0.041 | -0.0581 | No |
| 72 | ITGAM | integrin subunit alpha M [Source:HGNC Symbol;Acc:HGNC:6149] | 5820 | 0.007 | -0.0921 | No |
| 73 | RAC2 | Rac family small GTPase 2 [Source:HGNC Symbol;Acc:HGNC:9802] | 6098 | -0.013 | -0.1137 | No |
| 74 | RAF1 | "Raf-1 proto-oncogene, serine/threonine kinase [Source:HGNC Symbol;Acc:HGNC:9829]" | 6187 | -0.019 | -0.1203 | No |
| 75 | BDKRB2 | bradykinin receptor B2 [Source:HGNC Symbol;Acc:HGNC:1030] | 6276 | -0.026 | -0.1268 | No |
| 76 | ARHGEF12 | Rho guanine nucleotide exchange factor 12 [Source:HGNC Symbol;Acc:HGNC:14193] | 6587 | -0.049 | -0.1503 | No |
| 77 | PIP5K1C | phosphatidylinositol-4-phosphate 5-kinase type 1 gamma [Source:HGNC Symbol;Acc:HGNC:8996] | 6601 | -0.050 | -0.1505 | No |
| 78 | WAS | WASP actin nucleation promoting factor [Source:HGNC Symbol;Acc:HGNC:12731] | 6666 | -0.054 | -0.1546 | No |
| 79 | MAPK1 | mitogen-activated protein kinase 1 [Source:HGNC Symbol;Acc:HGNC:6871] | 6853 | -0.068 | -0.1681 | No |
| 80 | PDGFRB | platelet derived growth factor receptor beta [Source:HGNC Symbol;Acc:HGNC:8804] | 6914 | -0.073 | -0.1716 | No |
| 81 | MSN | moesin [Source:HGNC Symbol;Acc:HGNC:7373] | 6919 | -0.074 | -0.1707 | No |
| 82 | ARPC5L | actin related protein 2/3 complex subunit 5 like [Source:HGNC Symbol;Acc:HGNC:23366] | 7023 | -0.082 | -0.1774 | No |
| 83 | ITGAV | integrin subunit alpha V [Source:HGNC Symbol;Acc:HGNC:6150] | 7067 | -0.085 | -0.1794 | No |
| 84 | DIAPH1 | diaphanous related formin 1 [Source:HGNC Symbol;Acc:HGNC:2876] | 7072 | -0.086 | -0.1783 | No |
| 85 | MYL7 | myosin light chain 7 [Source:HGNC Symbol;Acc:HGNC:21719] | 7137 | -0.091 | -0.1818 | No |
| 86 | FGF2 | fibroblast growth factor 2 [Source:HGNC Symbol;Acc:HGNC:3676] | 7183 | -0.095 | -0.1837 | No |
| 87 | RAC1 | Rac family small GTPase 1 [Source:HGNC Symbol;Acc:HGNC:9801] | 7192 | -0.096 | -0.1827 | No |
| 88 | APC2 | APC regulator of WNT signaling pathway 2 [Source:HGNC Symbol;Acc:HGNC:24036] | 7271 | -0.101 | -0.1871 | No |
| 89 | GNA12 | G protein subunit alpha 12 [Source:HGNC Symbol;Acc:HGNC:4380] | 7294 | -0.102 | -0.1872 | No |
| 90 | NRAS | "NRAS proto-oncogene, GTPase [Source:HGNC Symbol;Acc:HGNC:7989]" | 7300 | -0.102 | -0.1858 | No |
| 91 | PTK2 | protein tyrosine kinase 2 [Source:HGNC Symbol;Acc:HGNC:9611] | 7315 | -0.103 | -0.1852 | No |
| 92 | FGFR2 | fibroblast growth factor receptor 2 [Source:HGNC Symbol;Acc:HGNC:3689] | 7374 | -0.108 | -0.1879 | No |
| 93 | PIK3CD | "phosphatidylinositol-4,5-bisphosphate 3-kinase catalytic subunit delta [Source:HGNC Symbol;Acc:HGNC:8977]" | 7405 | -0.111 | -0.1884 | No |
| 94 | ACTN3 | actinin alpha 3 [Source:HGNC Symbol;Acc:HGNC:165] | 7557 | -0.124 | -0.1982 | No |
| 95 | PAK5 | p21 (RAC1) activated kinase 5 [Source:HGNC Symbol;Acc:HGNC:15916] | 7558 | -0.124 | -0.1961 | No |
| 96 | FGF3 | fibroblast growth factor 3 [Source:HGNC Symbol;Acc:HGNC:3681] | 7637 | -0.130 | -0.2001 | No |
| 97 | VCL | vinculin [Source:HGNC Symbol;Acc:HGNC:12665] | 7687 | -0.133 | -0.2017 | No |
| 98 | PIK3CA | "phosphatidylinositol-4,5-bisphosphate 3-kinase catalytic subunit alpha [Source:HGNC Symbol;Acc:HGNC:8975]" | 7733 | -0.136 | -0.2029 | No |
| 99 | PAK2 | p21 (RAC1) activated kinase 2 [Source:HGNC Symbol;Acc:HGNC:8591] | 7762 | -0.139 | -0.2028 | No |
| 100 | ITGA9 | integrin subunit alpha 9 [Source:HGNC Symbol;Acc:HGNC:6145] | 7785 | -0.140 | -0.2022 | No |
| 101 | ITGA2 | integrin subunit alpha 2 [Source:HGNC Symbol;Acc:HGNC:6137] | 7857 | -0.145 | -0.2053 | No |
| 102 | ITGB7 | integrin subunit beta 7 [Source:HGNC Symbol;Acc:HGNC:6162] | 7873 | -0.146 | -0.2040 | No |
| 103 | ARHGEF6 | Rac/Cdc42 guanine nucleotide exchange factor 6 [Source:HGNC Symbol;Acc:HGNC:685] | 7900 | -0.147 | -0.2036 | No |
| 104 | FGFR1 | fibroblast growth factor receptor 1 [Source:HGNC Symbol;Acc:HGNC:3688] | 7905 | -0.148 | -0.2014 | No |
| 105 | PIK3CG | "phosphatidylinositol-4,5-bisphosphate 3-kinase catalytic subunit gamma [Source:HGNC Symbol;Acc:HGNC:8978]" | 7953 | -0.152 | -0.2025 | No |
| 106 | HRAS | "HRas proto-oncogene, GTPase [Source:HGNC Symbol;Acc:HGNC:5173]" | 7977 | -0.153 | -0.2018 | No |
| 107 | MYH14 | myosin heavy chain 14 [Source:HGNC Symbol;Acc:HGNC:23212] | 8032 | -0.158 | -0.2034 | No |
| 108 | CHRM3 | cholinergic receptor muscarinic 3 [Source:HGNC Symbol;Acc:HGNC:1952] | 8034 | -0.158 | -0.2008 | No |
| 109 | SOS2 | SOS Ras/Rho guanine nucleotide exchange factor 2 [Source:HGNC Symbol;Acc:HGNC:11188] | 8058 | -0.160 | -0.1999 | No |
| 110 | ARAF | "A-Raf proto-oncogene, serine/threonine kinase [Source:HGNC Symbol;Acc:HGNC:646]" | 8092 | -0.163 | -0.1997 | No |
| 111 | BRAF | "B-Raf proto-oncogene, serine/threonine kinase [Source:HGNC Symbol;Acc:HGNC:1097]" | 8171 | -0.169 | -0.2030 | No |
| 112 | PAK3 | p21 (RAC1) activated kinase 3 [Source:HGNC Symbol;Acc:HGNC:8592] | 8266 | -0.176 | -0.2074 | No |
| 113 | IQGAP1 | IQ motif containing GTPase activating protein 1 [Source:HGNC Symbol;Acc:HGNC:6110] | 8276 | -0.177 | -0.2052 | No |
| 114 | ACTN1 | actinin alpha 1 [Source:HGNC Symbol;Acc:HGNC:163] | 8385 | -0.184 | -0.2106 | No |
| 115 | F2R | coagulation factor II thrombin receptor [Source:HGNC Symbol;Acc:HGNC:3537] | 8447 | -0.190 | -0.2122 | No |
| 116 | MYH9 | myosin heavy chain 9 [Source:HGNC Symbol;Acc:HGNC:7579] | 8463 | -0.191 | -0.2101 | No |
| 117 | GSN | gelsolin [Source:HGNC Symbol;Acc:HGNC:4620] | 8507 | -0.194 | -0.2102 | No |
| 118 | IQGAP2 | IQ motif containing GTPase activating protein 2 [Source:HGNC Symbol;Acc:HGNC:6111] | 8598 | -0.201 | -0.2139 | No |
| 119 | RHOA | ras homolog family member A [Source:HGNC Symbol;Acc:HGNC:667] | 8601 | -0.201 | -0.2107 | No |
| 120 | ARHGEF7 | Rho guanine nucleotide exchange factor 7 [Source:HGNC Symbol;Acc:HGNC:15607] | 8746 | -0.212 | -0.2184 | No |
| 121 | DIAPH3 | diaphanous related formin 3 [Source:HGNC Symbol;Acc:HGNC:15480] | 8758 | -0.213 | -0.2157 | No |
| 122 | PIK3R3 | phosphoinositide-3-kinase regulatory subunit 3 [Source:HGNC Symbol;Acc:HGNC:8981] | 8801 | -0.217 | -0.2154 | No |
| 123 | PIK3R5 | phosphoinositide-3-kinase regulatory subunit 5 [Source:HGNC Symbol;Acc:HGNC:30035] | 8804 | -0.217 | -0.2119 | No |
| 124 | ARHGAP35 | Rho GTPase activating protein 35 [Source:HGNC Symbol;Acc:HGNC:4591] | 8835 | -0.220 | -0.2105 | No |
| 125 | CSK | C-terminal Src kinase [Source:HGNC Symbol;Acc:HGNC:2444] | 9056 | -0.236 | -0.2238 | Yes |
| 126 | PAK6 | p21 (RAC1) activated kinase 6 [Source:HGNC Symbol;Acc:HGNC:16061] | 9068 | -0.237 | -0.2207 | Yes |
| 127 | ITGA6 | integrin subunit alpha 6 [Source:HGNC Symbol;Acc:HGNC:6142] | 9112 | -0.240 | -0.2200 | Yes |
| 128 | MOS | "MOS proto-oncogene, serine/threonine kinase [Source:HGNC Symbol;Acc:HGNC:7199]" | 9138 | -0.243 | -0.2179 | Yes |
| 129 | MRAS | muscle RAS oncogene homolog [Source:HGNC Symbol;Acc:HGNC:7227] | 9158 | -0.244 | -0.2153 | Yes |
| 130 | RRAS2 | RAS related 2 [Source:HGNC Symbol;Acc:HGNC:17271] | 9165 | -0.245 | -0.2116 | Yes |
| 131 | GIT1 | GIT ArfGAP 1 [Source:HGNC Symbol;Acc:HGNC:4272] | 9236 | -0.251 | -0.2129 | Yes |
| 132 | RDX | radixin [Source:HGNC Symbol;Acc:HGNC:9944] | 9239 | -0.251 | -0.2088 | Yes |
| 133 | ITGA10 | integrin subunit alpha 10 [Source:HGNC Symbol;Acc:HGNC:6135] | 9296 | -0.256 | -0.2089 | Yes |
| 134 | DIAPH2 | diaphanous related formin 2 [Source:HGNC Symbol;Acc:HGNC:2877] | 9402 | -0.264 | -0.2127 | Yes |
| 135 | ITGB8 | integrin subunit beta 8 [Source:HGNC Symbol;Acc:HGNC:6163] | 9442 | -0.268 | -0.2113 | Yes |
| 136 | ARPC4 | actin related protein 2/3 complex subunit 4 [Source:HGNC Symbol;Acc:HGNC:707] | 9522 | -0.276 | -0.2128 | Yes |
| 137 | ITGAL | integrin subunit alpha L [Source:HGNC Symbol;Acc:HGNC:6148] | 9526 | -0.276 | -0.2084 | Yes |
| 138 | ITGB6 | integrin subunit beta 6 [Source:HGNC Symbol;Acc:HGNC:6161] | 9614 | -0.284 | -0.2104 | Yes |
| 139 | FGF17 | fibroblast growth factor 17 [Source:HGNC Symbol;Acc:HGNC:3673] | 9693 | -0.291 | -0.2117 | Yes |
| 140 | ITGA7 | integrin subunit alpha 7 [Source:HGNC Symbol;Acc:HGNC:6143] | 9724 | -0.294 | -0.2091 | Yes |
| 141 | FGF6 | fibroblast growth factor 6 [Source:HGNC Symbol;Acc:HGNC:3684] | 9739 | -0.296 | -0.2052 | Yes |
| 142 | SCIN | scinderin [Source:HGNC Symbol;Acc:HGNC:21695] | 9742 | -0.296 | -0.2004 | Yes |
| 143 | LIMK1 | LIM domain kinase 1 [Source:HGNC Symbol;Acc:HGNC:6613] | 9746 | -0.296 | -0.1956 | Yes |
| 144 | SSH1 | slingshot protein phosphatase 1 [Source:HGNC Symbol;Acc:HGNC:30579] | 9748 | -0.296 | -0.1907 | Yes |
| 145 | LIMK2 | LIM domain kinase 2 [Source:HGNC Symbol;Acc:HGNC:6614] | 9812 | -0.302 | -0.1905 | Yes |
| 146 | PXN | paxillin [Source:HGNC Symbol;Acc:HGNC:9718] | 9845 | -0.305 | -0.1879 | Yes |
| 147 | PIP5K1A | phosphatidylinositol-4-phosphate 5-kinase type 1 alpha [Source:HGNC Symbol;Acc:HGNC:8994] | 9851 | -0.306 | -0.1831 | Yes |
| 148 | BAIAP2 | BAR/IMD domain containing adaptor protein 2 [Source:HGNC Symbol;Acc:HGNC:947] | 9892 | -0.311 | -0.1810 | Yes |
| 149 | MAP2K2 | mitogen-activated protein kinase kinase 2 [Source:HGNC Symbol;Acc:HGNC:6842] | 9932 | -0.315 | -0.1788 | Yes |
| 150 | FGF4 | fibroblast growth factor 4 [Source:HGNC Symbol;Acc:HGNC:3682] | 9958 | -0.317 | -0.1754 | Yes |
| 151 | PAK4 | p21 (RAC1) activated kinase 4 [Source:HGNC Symbol;Acc:HGNC:16059] | 10004 | -0.322 | -0.1735 | Yes |
| 152 | ITGB4 | integrin subunit beta 4 [Source:HGNC Symbol;Acc:HGNC:6158] | 10159 | -0.337 | -0.1800 | Yes |
| 153 | MAP2K1 | mitogen-activated protein kinase kinase 1 [Source:HGNC Symbol;Acc:HGNC:6840] | 10197 | -0.340 | -0.1771 | Yes |
| 154 | FGF22 | fibroblast growth factor 22 [Source:HGNC Symbol;Acc:HGNC:3679] | 10261 | -0.347 | -0.1762 | Yes |
| 155 | FGF12 | fibroblast growth factor 12 [Source:HGNC Symbol;Acc:HGNC:3668] | 10294 | -0.350 | -0.1729 | Yes |
| 156 | ITGB3 | integrin subunit beta 3 [Source:HGNC Symbol;Acc:HGNC:6156] | 10310 | -0.352 | -0.1681 | Yes |
| 157 | FGF21 | fibroblast growth factor 21 [Source:HGNC Symbol;Acc:HGNC:3678] | 10341 | -0.355 | -0.1645 | Yes |
| 158 | APC | APC regulator of WNT signaling pathway [Source:HGNC Symbol;Acc:HGNC:583] | 10440 | -0.366 | -0.1660 | Yes |
| 159 | INSRR | insulin receptor related receptor [Source:HGNC Symbol;Acc:HGNC:6093] | 10459 | -0.369 | -0.1612 | Yes |
| 160 | ACTN4 | actinin alpha 4 [Source:HGNC Symbol;Acc:HGNC:166] | 10472 | -0.370 | -0.1559 | Yes |
| 161 | MYL12B | myosin light chain 12B [Source:HGNC Symbol;Acc:HGNC:29827] | 10505 | -0.374 | -0.1521 | Yes |
| 162 | KRAS | "KRAS proto-oncogene, GTPase [Source:HGNC Symbol;Acc:HGNC:6407]" | 10544 | -0.377 | -0.1487 | Yes |
| 163 | ROCK2 | Rho associated coiled-coil containing protein kinase 2 [Source:HGNC Symbol;Acc:HGNC:10252] | 10648 | -0.388 | -0.1503 | Yes |
| 164 | CHRM5 | cholinergic receptor muscarinic 5 [Source:HGNC Symbol;Acc:HGNC:1954] | 10725 | -0.396 | -0.1496 | Yes |
| 165 | TMSB4X | thymosin beta 4 X-linked [Source:HGNC Symbol;Acc:HGNC:11881] | 10741 | -0.398 | -0.1441 | Yes |
| 166 | FGD1 | "FYVE, RhoGEF and PH domain containing 1 [Source:HGNC Symbol;Acc:HGNC:3663]" | 10892 | -0.416 | -0.1489 | Yes |
| 167 | BDKRB1 | bradykinin receptor B1 [Source:HGNC Symbol;Acc:HGNC:1029] | 10951 | -0.424 | -0.1463 | Yes |
| 168 | PIK3R2 | phosphoinositide-3-kinase regulatory subunit 2 [Source:HGNC Symbol;Acc:HGNC:8980] | 10956 | -0.425 | -0.1394 | Yes |
| 169 | ITGA8 | integrin subunit alpha 8 [Source:HGNC Symbol;Acc:HGNC:6144] | 11046 | -0.437 | -0.1390 | Yes |
| 170 | PIK3R1 | phosphoinositide-3-kinase regulatory subunit 1 [Source:HGNC Symbol;Acc:HGNC:8979] | 11099 | -0.443 | -0.1356 | Yes |
| 171 | NCKAP1 | NCK associated protein 1 [Source:HGNC Symbol;Acc:HGNC:7666] | 11256 | -0.465 | -0.1401 | Yes |
| 172 | PPP1R12A | protein phosphatase 1 regulatory subunit 12A [Source:HGNC Symbol;Acc:HGNC:7618] | 11259 | -0.465 | -0.1324 | Yes |
| 173 | WASF1 | WASP family member 1 [Source:HGNC Symbol;Acc:HGNC:12732] | 11387 | -0.484 | -0.1342 | Yes |
| 174 | VAV2 | vav guanine nucleotide exchange factor 2 [Source:HGNC Symbol;Acc:HGNC:12658] | 11413 | -0.489 | -0.1279 | Yes |
| 175 | ITGAX | integrin subunit alpha X [Source:HGNC Symbol;Acc:HGNC:6152] | 11526 | -0.505 | -0.1282 | Yes |
| 176 | ITGA2B | integrin subunit alpha 2b [Source:HGNC Symbol;Acc:HGNC:6138] | 11645 | -0.524 | -0.1287 | Yes |
| 177 | PPP1CB | protein phosphatase 1 catalytic subunit beta [Source:HGNC Symbol;Acc:HGNC:9282] | 11737 | -0.539 | -0.1267 | Yes |
| 178 | SOS1 | SOS Ras/Rac guanine nucleotide exchange factor 1 [Source:HGNC Symbol;Acc:HGNC:11187] | 12041 | -0.603 | -0.1404 | Yes |
| 179 | ROCK1 | Rho associated coiled-coil containing protein kinase 1 [Source:HGNC Symbol;Acc:HGNC:10251] | 12046 | -0.605 | -0.1305 | Yes |
| 180 | PDGFA | platelet derived growth factor subunit A [Source:HGNC Symbol;Acc:HGNC:8799] | 12104 | -0.621 | -0.1245 | Yes |
| 181 | EGFR | epidermal growth factor receptor [Source:HGNC Symbol;Acc:HGNC:3236] | 12109 | -0.622 | -0.1143 | Yes |
| 182 | ABI2 | abl interactor 2 [Source:HGNC Symbol;Acc:HGNC:24011] | 12114 | -0.624 | -0.1041 | Yes |
| 183 | WASL | WASP like actin nucleation promoting factor [Source:HGNC Symbol;Acc:HGNC:12735] | 12207 | -0.647 | -0.1004 | Yes |
| 184 | PIKFYVE | "phosphoinositide kinase, FYVE-type zinc finger containing [Source:HGNC Symbol;Acc:HGNC:23785]" | 12347 | -0.690 | -0.0997 | Yes |
| 185 | CFL1 | cofilin 1 [Source:HGNC Symbol;Acc:HGNC:1874] | 12352 | -0.691 | -0.0884 | Yes |
| 186 | PDGFB | platelet derived growth factor subunit B [Source:HGNC Symbol;Acc:HGNC:8800] | 12389 | -0.704 | -0.0793 | Yes |
| 187 | ITGA5 | integrin subunit alpha 5 [Source:HGNC Symbol;Acc:HGNC:6141] | 12474 | -0.737 | -0.0735 | Yes |
| 188 | ITGB1 | integrin subunit beta 1 [Source:HGNC Symbol;Acc:HGNC:6153] | 12566 | -0.780 | -0.0675 | Yes |
| 189 | ACTG1 | actin gamma 1 [Source:HGNC Symbol;Acc:HGNC:144] | 12613 | -0.806 | -0.0575 | Yes |
| 190 | ARHGEF1 | Rho guanine nucleotide exchange factor 1 [Source:HGNC Symbol;Acc:HGNC:681] | 12673 | -0.846 | -0.0479 | Yes |
| 191 | CDC42 | cell division cycle 42 [Source:HGNC Symbol;Acc:HGNC:1736] | 12681 | -0.852 | -0.0341 | Yes |
| 192 | ACTB | actin beta [Source:HGNC Symbol;Acc:HGNC:132] | 12757 | -0.920 | -0.0245 | Yes |
| 193 | FN1 | fibronectin 1 [Source:HGNC Symbol;Acc:HGNC:3778] | 12820 | -1.005 | -0.0124 | Yes |
| 194 | GNA13 | G protein subunit alpha 13 [Source:HGNC Symbol;Acc:HGNC:4381] | 12862 | -1.103 | 0.0030 | Yes |
Table: GSEA details [plain text format]

  

Fig 2: KEGG\_REGULATION\_OF\_ACTIN\_CYTOSKELETON      
 Blue-Pink O' Gram in the Space of the Analyzed GeneSet

  

Fig 3: KEGG\_REGULATION\_OF\_ACTIN\_CYTOSKELETON: Random ES distribution      
 Gene set null distribution of ES for **KEGG\_REGULATION\_OF\_ACTIN\_CYTOSKELETON**
